# To the Women Readers

**Published:** 1912-10

**Authors:** 


### To the Women Readers.

*Dear Friends:*

I hope you will be pleased to learn that we are going to have a "Question Box" for the benefit of the readers of the *"Red Back."* There will be an advisory board, composed in each instance of specialists, to whom these questions will be submitted. There will be a nerve specialist; an eye, ear, nose and throat specialist; a stomach specialist; a specialist of children's diseases; a hookworm specialist; a specialist for weak-minded children; a specialist of women's diseases to whom you may come with the most delicate of questions, because the "woman specialist" is a woman who understands and loves her suffering sisters as only those who *know* can love. So if there is anything you want to know just address it to the specialist under whose supervision it belongs and send it to the *"Red Back,"* 204 East Tenth street, Austin, Texas.

Next month I am going to have a delightful surprise for you. No, I am not going to tell you one word, except that it concerns one of the most loved and prominent club women in Texas. Just you look out for the "Woman's Department" next month.

Ask your doctor why so many joyous, healthy girls become invalids after marriage.

Ask your doctor why so many children are born blind.

Ask your doctor to give you his point of view as regards the "divorce evil."

Ask your doctor, and be *sure* that he answers your question, why so many women die of cancer of the womb.

